# Supplementary material for: Enhancing the knowledge of parents on child health using eLearning in a government school in the semi-rural community of Karachi, Pakistan
Source: PLOS Glob Public Health. 2022 Jun 15;2(6):e0000500. doi: 10.1371/journal.pgph.0000500 (PMC10022312; doi:10.1371/journal.pgph.0000500)
Supplement: S1 Text — (DOC) [file pgph.0000500.s001.doc]

**PRE TEST QUESTIONS IN ROMAN URDU ہیلتھ سیشن سے پہلے ٹیسٹ**

Q1: Measles is an infection caused by measles virus?

سوال نمبر1 ۔ کیا خسرہ کی بیماری خسارے کے جرا‌ث‌یم سے ہوتی ہے؟ A. True صحیح B. False غلط

Q2: Measles is not contagious?

سوال نمبر2 ۔ خسرہ پھلنے والی بیماری نہیں ہیں ؟

A. True صحیح B. False غلط

Q3: What are the initial symptoms of measles?

سوال نمبر3 ۔ خسرہ کے ابتداء علامات کیا ہیں ؟

- Vomiting and abdominal pain الٹیاں اور پیٹ میں درد 1
- Fever, cough, conjunctivitis آنکھیں سورخ ۔ کھانسی بخار ۔ 2
- Headache and neck pain سر درد اور گردن درد 3

Q4: What is the most common complication of measles? ( Chاoose the best answer)

سوال نمبر4 ۔ خسارے کا سب سے زیادہ عام پیچیدہ کیا ہے؟

1. Ear infection کان کے مسائل 1
2. Skin infection جلد کی انفیکشن 2
3. Pneumonia نمونیہ 3
4. Brain infection دماغ کی بیماری 4

Q5: At what age/ages measles vaccination given?

سوال نمبر5 ۔ کونسی عمر میں بچے کو خسارے کا ٹیکہ لگاتے ہے؟

1. 9 months only صرف 9 ماہ میں 1
2. 9 months and 15 months ماہ میں 15 اور 9 ماہ2
3. 15 months only صرف 15 ماہ میں 3
4. 6 months and 12 ماہ میں 12 اور 6 ماہ4

Q6: From where rash of measles starts?

سوال نمبر6 ۔ • خسرے کے دانے جسم کے کونسے حصے سے شروع ہوتے ہيں؟

1. Legs ٹانگوں پہ 1
2. Back پیٹھ پہ 2
3. Behind the ear کان کے پیچھے 3
4. Abdomen پہ پیٹ 4
