# Supplementary material for: Enhancing the knowledge of parents on child health using eLearning in a government school in the semi-rural community of Karachi, Pakistan
Source: PLOS Glob Public Health. 2022 Jun 15;2(6):e0000500. doi: 10.1371/journal.pgph.0000500 (PMC10022312; doi:10.1371/journal.pgph.0000500)
Supplement: Attachment — Submitted filename: Response to reviewers.docx [file pgph.0000500.s002.docx]

Comment: PGPH-D-21-00889

Empowering parents of school children using eLearning in a government school of semi-rural community of Karachi, Pakistan Dr. Saleema Gulzar

Dear Dr. Gulzar,

We've checked your submission and before we can proceed, we need you to address the following issues:

1. We see that your study includes live participants. Please update your manuscript file to include a statement that formal consent was obtained (must state whether verbal/written).

**Response:** Thank you for your feedback. In line 96 of the manuscript. We have added, "Written informed consent was formally obtained from all participating individuals."
