# Supplementary material for: Enhancing the knowledge of parents on child health using eLearning in a government school in the semi-rural community of Karachi, Pakistan
Source: PLOS Glob Public Health. 2022 Jun 15;2(6):e0000500. doi: 10.1371/journal.pgph.0000500 (PMC10022312; doi:10.1371/journal.pgph.0000500)
Supplement: Attachment — Submitted filename: Respose to reviewersdocx.docx [file pgph.0000500.s003.docx]

Rebuttal letter

To,

The Editor,

PLOS global health,

**Subject: response to reviewers’ comments**

**Dear Sir,**

I would like to express my gratitude to you for sharing extensive feedback to improve the submitted manuscript No. PGPH-D-21-00889, “Empowering parents of school children using eLearning in a government school of semi-rural community of Karachi, Pakistan.

Below are the point wise responses based on the comments received from reviewers.

| Reviewer’s comments | Author comments | Page number |
| --- | --- | --- |
| Reviewer #1:  **1. Title:**  a. Does not reflect what has been discussed in the introduction i.e., health education.  b. Empowering parents of …. What???  **2. Abstract:**  a. Introductory sentence should highlight the importance of school health programme rather than PHC and health education in general.  b. Rewrite the keywords according to the MeSH terms.  **3- Introduction:**  a. Poorly written, lack of flow and key topics were not discussed.  b. For example, impact of eLearning-based school health education programs was not discussed. Evidence from regional countries should be documented.  c. Common health problems and issues of children in different countries were not adequately discussed.  d. The need for health education and the present status of health education in primary and secondary schools in Pakistan/regional countries require further discussion.  **4. Methods:**  a. Is this a primary or secondary school?  b. Who developed and finalized the questionnaire?  c. Who conducted the FGDs? Are they trained and/or expert in conducting FGDs?  d. Were the researchers trained/experts to conduct data analysis?  **5.Results:**  a. Sub-themes (Table 2) were not discussed clearly.  b. The word “empowering” was mentioned in the title; However, it was not mentioned/discussed either in the introduction, results, or discussion sections  **6. Discussion** a. The key findings need to support by evidence/study findings from Pakistan, South-East Asian and developing countries in addition to developed countries.  **7. Conclusion** a. Not included b. Summarize the key findings and include specific recommendations based on the findings of your research. | Title has been modified as per the feedback.  Correction has been incorporated  Keywords adjusted  Correction has been made in introduction as suggested.  The school offers primary and secondary education till class VIII.  Suggested points are incorporated in material and methods section.  Subthemes have been elaborated as suggested.  The title has been modified  The key findings are supported by relevant literature  Conclusion is added | Page number 1 (line 1-3)  Page number 2 (line 23-34, 37-39)  Page number 3 (line 46)  Page number 4 and 5 (line 63-66, 69-71, 73-85, 87-99, 101-108)  Page number 6 and 7 (line 111-114, 116-124, 126-130, 139-147, 151-155, 157-162)  Page number 8 (line 167, 168)  Page number 1 (line 1-3)  Page number 15-17 (277-283, 285-289, 291, 292, 296, 299-302, 312-314, 316-318, 320-325).  Page number 17 and 18 (line 342-349) |
| **Reviewer #2:**  I think this manuscript is a good concept and will add to the growing body of knowledge on virtual learning. It is also uniquely targeting parents.  I have however noted the following from my point of view:  line 83 - started in a semi-urban to replace started a semi-urban (minor typo I guess).  line 98- In the methods section, I would have expected to see the population of students stated and the target population of parents calculated. I am of the view teachers should have been included in the initial formal meetings as key stakeholders.  line 99- I think the health care professionals who reviewed the baseline data should be better defined in terms of number and expertise.  line 101 - I am not sure how this worked out. Parents preferred time or agreed time? I would have expected to see the number invited for the sessions to compare with the number participating.  line 104- You may wish to elaborate more about the virtual software and situate it on a specific type of e learning. It may be important to define e learning in the introduction and specify the type used in this study.  lines 111/112 - please verify if the school health nurse was female before using the pronoun 'she', otherwise the statement should be gender neutral.  lines 118-121 - How many FGDs were conducted? what was the composition of each group in terms of homogeneity and heterogeneity (were parents, community leader and teachers mixed or separated) in the groups? How was FGD recorded?  line 211- Please write acronyms in full when used for the first time and provide the acronym in parentheses before subsequent usage.  On a general note, the study did not have examples from similar income country context for comparisons. It may also be helpful to state the availability and accessibility of internet to the parents and target communities. The ownership an access to appropriate electronic devices to join in the virtual learning are also important consideration.  Future studies on this subject matter should consider having schoolteachers on the research team and considering utilizing an implementation science approach | Point taken and adjusted.  Feedback integrated  All the feedback are integrated  All the feedback are integrated  All the feedback are integrated | Page number 4 (line 80)  Page number 6 (line 121-124)  Page number 6 (line 124-126)  Page number 6 (line 119-121, 127)  Page number 5 and 6 (line 101-106, 129)  Page number 7 (line 145)  Page number 8 and 9 (line 151-155)  Page number 15 (lines 287, 288)  Page number 13 (line 247-255)  Page number 18 (line 348) |
